# PROTOCOL: Studies of the effectiveness of interventions to improve the welfare of those affected by, and at risk of, homelessness in high‐income countries: An evidence and gap map

**DOI:** 10.1002/cl2.1069

**Published:** 2019-12-23

**Authors:** Howard White, Ashrita Saran, Ben Fowler, Audrey Portes, Suzanne Fitzpatrick, Ligia Teixeira

**Affiliations:** ^1^ Campbell Collaboration Global Development Network New Delhi India; ^2^ Centre for Homelessness Impact CRISIS London UK; ^3^ Campbell Collaboration Folkehelseinstituttet Oslo Norway; ^4^ Institute for Social Policy, Housing, Environment and Real Estate Herriot Watt University Edinburgh UK

## Abstract

Homelessness – people living on the street, in temporary accommodation, or at risk of losing their homes – is a persistent problem across the developed world. Policies and programmes to tackle homelessness should be informed by evidence of effectiveness. This is the protocol for an evidence and gap map for studies of the effectiveness of interventions to improve the welfare of those experiencing homelessness or at risk of homelessness. We proposed a comprehensive search for studies, with systematic screening, coding and reporting. The available studies will be presented in an online interactive map together with a supporting report.

## 1 BACKGROUND

### 1.1 The problem, condition or issue

Weak economic performance and rising property costs increase the numbers of those homeless, at risk of becoming homeless and living in inadequate housing. Estimates of “core homelessness” in 2016 stood at around 160,000 households in Great Britain.

Homelessness, even of short durations, can result in socioeconomic exclusion with reduced access to a range of social services and reduced employment possibilities. People experiencing homeliness have worse health outcomes, and there is a mutual relationship between homelessness and other social disadvantages, such as mental health problems and substance abuse.

Effective interventions are, therefore, required to place and keep people in stable housing, and address the health and wider support needs of all people experiencing or at risk of homelessness. There is a range of interventions to try to prevent homelessness and to increase housing stability. However, the evidence base of studies of the effectiveness of these interventions is thought to be uneven by both study design and geography, with most studies being conducted in North America.

Development of the map will support efforts to tackle socioeconomic exclusion, and sustained deprivation and inequality. It will support related research initiatives such as Inclusion Health (Luchenski et al., 2018). And importantly the maps will support a suite of evidence tools produced by the Centre for Homelessness Impact (CHI; http://homelessnessimpact.org).

### 1.2 Scope of the EGM

*Full name*: An Evidence and Gap Map of Studies of the Effectiveness for Those Affected by and at Risk of Homelessness in High‐Income Countries.

*Short name*: Homelessness: an evidence and gap map

Homelessness is broadly defined to include not only those sleeping rough. Those experiencing homelessness are those who have no accommodation and so sleep on the street (sleeping rough) or are in temporary (i.e., transitional), insecure or poor‐quality housing (European Commission, no date). People in temporary shelters or other transitional accommodation are still considered homeless. Those at risk of homelessness may currently be in satisfactory accommodation but at risk of losing it—for example, because of loss of employment or other income sources.

The interventions, which are listed below, are interventions whose main purpose is to improve the welfare of those experiencing or are at risk of homelessness.

### 1.3 Conceptual framework of the EGM

**Figure 1:**
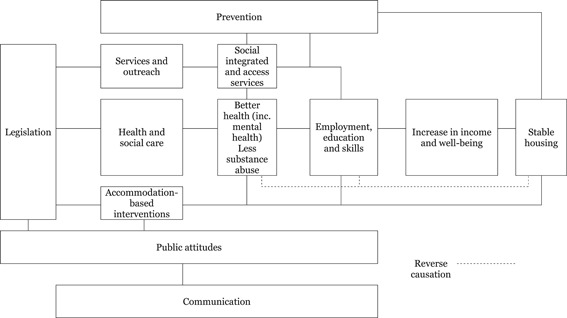
Overview of logic model for homelessness interventions

Figure 1 shows the logic model for the interventions and how they link to the major outcomes. This does not provide a detailed theory of change of how specific interventions are meant to work but rather provides an overview of the policy space covered by the evidence map and how those parts fit together.

Key features are as follows.

1. Legislation sets the context affecting services, care and accommodation.
2. Public opinion, which is affected by advocacy and communication, affects legislation and provision of interventions such as accommodation.
3. Services and outreach and social care can improve health and reduce substance abuse, thus allowing access to education and skills training and so employment.
4. These can lead to increased income and so stable housing and improved wellbeing.
5. Providing accommodation can support stable housing which in turn supports health and employment prospects (the reverse causation shown by the dotted line).
6. Prevention enters into this causal chain at several points.
7. Interventions interact reflecting that clients often need multiple services.

### 1.4 Why it is important to develop the EGM

Currently, there is no single resource that allows policy makers, practitioners and researchers working to improve the welfare of those experiencing homelessness to access the available relevant evidence on which programmes work. The review team is working with the UK Centre for Homelessness Impact to develop the evidence architecture for the sector.

The CHI plans to become a “one stop shop” for evidence for policy makers and practitioners in the sector. As a first step, working with the Campbell Collaboration, the Centre is producing to two evidence maps of evidence on homelessness. This protocol is for the map of effectiveness studies of What Works to improve the Welfare of those Experiencing Homelessness. A second map will show implementation issues for such interventions as identified in process evaluations. The two maps together will comprise the largest single source globally of evidence on interventions for those experiencing and at risk of homelessness.

CHI aims to improve the welfare of people affected by homelessness by providing evidence‐based resources for policy makers and practitioners. The EGMs are the first part of that evidence architecture, and a building block for what will come next. The maps will identify the evidence to be used in the Centre's online evidence resources. And the maps will inform the future‐policy‐oriented research programme of the Centre.

In the coming years, CHI will be commissioning new studies to assess the effectiveness of programmes for those affected by homelessness. The map will inform the identification of priority areas where evidence is currently lacking, such as rigorous studies of the effectiveness of reconnection programmes or those being discharged from mental health or penal institutions.

### 1.5 Existing EGMs and relevant systematic reviews

We are aware of two other maps related to homelessness. One is being prepared by the Canadian Homelessness Health Network. That map has a narrower focus than ours, but we are sharing resources with the team to ensure consistency in coverage. A second, unpublished map, was produced by the Sax Institute for the New South Wales state government. That map, which includes only 16 studies, is narrower in scope than the proposed sector wide map we will produce.

There are a number of systematic reviews, all of which are narrower than the proposed map.

Most recent is a rapid evidence review undertaken by CRISIS. The review has a broad scope but limits the evidence being reviewed: 120 studies were identified as high quality of which 35 were analysed (SCIE, 2018). Munthe‐Kas et al. (2016) restrict their systematic review to studies that assess the impact of interventions on housing status. They include 43 studies but list around 100 more which report other outcomes. The systematic review by Altena, Brilleslijper‐Kater, and Wolf (2010) is restricted to homeless youth.

In addition, there are a number of more focused reviews. For example: (a) the systematic review by Bassuk, DeCandia, Tsertsvadze, and Richard (2014) assesses the impact of housing interventions on family homelessness; (b) Hwang, Tolomiczenko, Kouyoumdjian and Garner (2005) review the effectiveness of health interventions for homeless populations; and (c) Byrne, Montgomery, and Dichter (2013) report studies relate to homelessness amongst female veterans. There are three on‐going reviews registered with Campbell which have been identified on the basis of earlier editions of this map.

There are also prevalence reviews related to homelessness, especially related to mental health (e.g., Folsom and Jeste (2002) on schizophrenia, Hodgson, Shelton, van den Bree, & Los, 2013 on psychopathology, and Fazel, Khosla, Doll, & Geddes, 2008 on mental disorders in general). As these studies are not studies of effects, they are not relevant to this map.

## 2 OBJECTIVES

The proposed EGM will present studies of the effectiveness of these interventions across a range of outcome domains. Specifically, the objectives of the map are to:

1. develop a clear taxonomy of interventions and outcomes related to homelessness in high‐income countries;
2. map available systematic reviews and primary studies of the effectiveness of interventions for those experiencing homelessness and those at risk of homelessness, with an overview provided in a summary report;
3. provide database entries of included studies that summarise the intervention, context, study design and main findings.

## 3 METHODOLOGY

### 3.1 Defining EGMs

This evidence and gap map (EGM) is an effectiveness map in which the primary dimensions are the rows and columns of the map which are, respectively, intervention categories (and subcategories) and indicator domains (and subdomains). Secondary dimensions, such as country and target group will be included as filters.

### 3.2 EGM framework

The EGM framework will inform the inclusion and exclusion criteria of the EGM. Here, we describe the population, intervention, comparison, outcomes (indicators) and study designs (PICOS) for the map.

#### 3.2.1 Population

The population is individuals and families who are homeless or at risk of becoming homeless.

Population subgroups of interest are listed under filters.

#### 3.2.2 Intervention

Table 1 lists the intervention categories. Examples of programme names are given in brackets. These are listed to aid with search and coding. They will not appear in the subcategory label in the map. Some programmes are either multicomponent or straddle intervention subcategories. Examples are Housing First (congregate/scatter site; ACT/ICT) and Homeless Veterans’ Reintegration Program. Studies of these interventions can appear in more than one category. The map will have a searchable field (filter) for programme name where these programmes are not included in the intervention subcategories (see below on filters).

**Table 1:** Intervention categories and subcategories

Accommodation	Accommodation (excluding emergency accommodation) with minimal or no support services. This includes: community‐led housing; modular homes; private‐rented sector; social housing; temporary accommodation.
Accommodation with support services	Accommodation (excluding emergency accommodation) combined with some form of support services. This includes: housing first; rapid rehousing; supported accommodation and supported lodging; hostels; women's refuges.
Accommodation‐based support services	Accommodation (excluding emergency accommodation) based support services. This includes continuum of care/staircase; coordinated assessment; floating support; housing advice; landlord/tenant mediation; tenancy training.
Armed forces	Interventions targeted at people in the armed forces. This includes: induction and initial training; on‐going development and support; discharge from armed services.
Arts, sports and culture	Mainstream and specialist arts, sports and cultural activities.
Communication and campaigns	Communications and campaigns interventions. This includes: behavioural insights approaches; government information campaigns; lobbying; public influencing campaigns.
Crime and justice	Crime and justice interventions related to homelessness. This includes: courts; enforcement and criminalisation; policing; prison; probation and rehabilitation.
Direct donations and relief grants	Direct donations to people who are homeless or at risk of homelessness.
Education and skills and employment	Education and vocational training for adults.
Health and social care: mainstream	Mainstream health and social care interventions (e.g., in a regular doctor's surgery or hospital). This includes interventions in mainstream: adult social care; children in care services; physical health; substance misuse; mental health; discharge from health and social care; co‐location or embedded in mainstream service.
Health and social care: specialist	Specialist homelessness health and social care interventions (i.e., not within mainstream systems). This includes specialist: children in care services; psychologically informed environments; adult social care; physical health; substance misuse; mental health; case management/critical time intervention.
Public policy for housing and homelessness	General macro‐level housing or homelessness public policy/legislation. This includes: financing models; housing/planning policies; homelessness policies.
Public policy—other	Wider macro‐level public policy in nonhousing/homelessness areas. This includes: conditionality; crime, justice and the law; education; health and social care; immigration; integrating services; transport; welfare benefits.
Social relationships and community	Social relationship and community interventions. This includes: befriending, mentoring and coaching; family mediation; social/community networks.
Technology	Technology interventions. This includes: apps and websites; digital inclusion; systems.

#### 3.2.3 Indicators (outcomes)

The indicator domains are shown in Table 2. There are seven domains: (a) access to services, (b) crime/criminalisation, (c) housing stability, (d) health (including substance abuse), (e) employment and income, (f) capabilities and wellbeing, and (g) public attitudes and participation.

**Table 2:** Indicator domains

Domain	Subdomains
Access to services	Health care (primary, secondary and tertiary care)
	Welfare benefits
	Other services
Crime and justice	Arrest and imprisonment (including criminalisation of street living)
	Recidivism
	Victims of crime
Housing stability	Accommodation/housing status
	Satisfaction with housing
Health (inc substance abuse)	Abstinence from substance abuse
	Physical health and nutrition status
	Mental health status
Employment and income	Employment status (paid and unpaid work)
	Skills
	Earned income
	Forced labour/labour and sex work
Capabilities and wellbeing	Skills in self‐care management, safe community participation, food and money management
	Community engagement and social connectedness
	Overall wellbeing/quality of life
Public attitudes and participation	Public understanding of homelessness
	Support for interventions for homelessness
	Fundraising
	Public engagement in homelessness‐related activities
Cost	Cost effectiveness
	Savings
	Cost per participant

### 3.3 Criteria for including and excluding studies

#### 3.3.1 Types of study designs

This is a map of the effectiveness of interventions to improve the welfare of those experiencing, or at risk of, homelessness. Effectiveness studies are those using large and statistical designs to measure the impact of an intervention, or systematic reviews of such studies.

Given this purpose, the map will include experimental and nonexperimental impact evaluations with a design which controls for selection bias. The following designs will be included: RCTs, natural experiments, regression discontinuity, propensity score matching, difference in difference, instrumental variables, and other matching designs. Before versus after designs with no control group will not be included.

The map will also include systematic reviews of effects that include studies from high‐income countries.

Comparison: Studies with both active and passive controls will be included.

#### 3.3.2 Treatment of qualitative research

We do not plan to include qualitative research in this map. A separate map is being prepared that will include process evaluations.

#### 3.3.3 Types of settings

Studies will be from high‐income countries.

#### 3.3.4 Status of Studies

On‐going studies will be included. Status of studies will be a filter.

### 3.4 Search strategy and status of studies

The search strategy comprises both, where to look and how to look. This map is being produced in stages. The approach for both elements of the search strategy is described below.

On account of the need for early results for CHI the map is being produced in the following stages.

1. Stage 1 will map the approximately 140 studies identified by Munthe‐Kaas, Berg, and Blaasvær (2016) plus around 30 systematic reviews identified during scoping. This map was published in mid‐2018. In this case, the where and how to look for the search strategy are clear. They were limited but adopted so that stakeholders could see what the map looked like.
2. Stage 2 will map the results from the full database search, including both primary studies and systematic reviews. This search is described below. This map was published in March 2019.
3. Stage 3 will be the version of the map published in the Campbell Library. In addition to the above, we will (a) search additional websites for grey literature, (b) screen all included studies in included systematic reviews, (c) consult experts, and (d) screen submissions received in response to dissemination of the Stages 1 and 2 maps.

#### 3.4.1 Database search for Stages 2 and 3

The databases to be searched are as follows.

1. Academic databases
  - Econlit
  - The National Bureau of Economic Research (NBER)
  - Social Science Research Network (SSRN)
  - International Bibliography of Social Sciences (IBSS)
  - Applied Social Sciences Index and Abstracts (ASSIA)
  - Social Service Abstract
  - Embase
  - PubMed
  - PsycINFO
  - MEDLINE
  - WHO's Global Health Library
  - CABI's Global Health
  - ERIC
  - CINHAL
  - SCOPUS
  - Web of Science
  - EPPI Centre Evaluation Database of Education Research

2. Evidence and Gap Map Database
  - 3ie Evidence and Gap Map Repository
  - Global Evidence Mapping Initiative
  - Evidence‐Based Synthesis Program (Department of Veteran Affairs)

3. Systematic review databases
  - Swedish Agency for Health Technology Assessment and Assessment of Social Services
  - Collaboration for Environmental Evidence
  - Cochrane
  - Cochrane
  - Campbell
  - 3ie Systematic Review Database
  - Research for Development
  - Epistemonikos

4. Trials registries

AEA Social Science RCT Registry https://www.socialscienceregistry.org/.

Sample search terms are listed in Appendix A.

We will also undertake a more limited search of French, Spanish, Portuguese and Norwegian academic databases.

**Table:** 

French	Spanish	Portuguese	Norwegian
Scholar.google.fr			Scholar.google.no
Google.fr			Google.no
Cairn.info			
Persee.fr			

All titles and abstracts, and then full text, will be double screened, with a third‐party arbitrator in the event of disagreement.

#### 3.4.2 Grey literature and websites

In addition to electronic studies, we shall search and screen publications from the following websites.

Homeless Hub https://www.homelesshub.ca/

European observatory on homelessness https://www.feantsaresearch.org/en/publications

United State interagency council on homelessness http://www.usich.gov/

EThOS http://ethos.bl.uk/Home.do

WHO ICTRP http://apps.who.int/trialsearch/

Focus on Prevention http://www.preventionfocus.net/

Social Policy and Practice http://www.spandp.net/

10,000 home campaigns https://en.wikipedia.org/wiki/100,000_Homes_Campaign

Anti‐poverty committee https://en.wikipedia.org/wiki/Anti‐Poverty_Committee

Back on my feet https://en.wikipedia.org/wiki/Back_on_My_Feet_(non‐profit_organization)

Feantsa https://www.feantsa.org/

National Coalition Homeless https://nationalhomeless.org/

Homelessness Australia https://www.homelessnessaustralia.org.au/

Mission Australia https://www.missionaustralia.com.au/publications/position‐statements/homelessness

National Alliance to end homelessness https://endhomelessness.org/

Institute of global homelessness https://www.ighomelessness.org/

Homelessness link https://www.homeless.org.uk/

Crisis https://www.crisis.org.uk/about‐us/how‐we‐work/

Housing first https://housingfirsteurope.eu/about‐the‐hub/

Canadian Alliance to end homelessness https://housingfirsteurope.eu/about‐the‐hub/

Social work and policy institutes http://www.socialworkpolicy.org/research/homelessness.html

Association of housing advice services https://www.ahas.org.uk/

Centre point https://centrepoint.org.uk/

Homelessness trust funds https://housingtrustfundproject.org/htf‐elements/homeless‐trust‐funds/

Meliville charitable trust https://melvilletrust.org/category/resources‐reports/

Conrad H Hilton foundation https://www.hiltonfoundation.org/priorities/homelessness#resources

Abt Associates https://www.abtassociates.com/

Mathematica https://www.mathematica‐mpr.com/

American Institutes of Research https://www.air.org/

Rand https://www.rand.org/

MDRC https://www.mdrc.org/

We will also search Google and Scholar Google.

#### 3.4.3 Contacting researchers

We will send copies of the preliminary map to authors of included studies, which serves both a dissemination purpose and to invite submission of additional studies.

### 3.5 Data extraction, coding and management

Coding will be done independently by two coders, with a third‐party arbitrator in the event of disagreement.

*Coding of bibliographic information and intervention and study design and characteristics*

Full bibliographic information will be captured, along with the information necessary to construct the map (interventions, outcomes and filters). The coding form is given in Appendix B.

#### 3.5.1 Critical appraisal

Coding will also capture the data needed for critical appraisal of all included studies. Critical appraisal of primary studies shall be conducted using the tool contained in Appendix C. The quality of the included systematic reviews will be assessed using AMSTAR 2.

## 4 ANALYSIS AND PRESENTATION

### 4.1 Unit of Analyses

The unit of analysis is each paper. Each entry in the map is a report or paper.

It is possible (indeed likely for public health) that there are multiple papers for a single study. If this occurs as there are different versions of the same paper, then only the latest or most complete version will be used in the map. However, if different papers report different analyses—for example, on different outcomes or for different population subgroups—then each such paper is included in the map. Hence, in principle, there may be multiple entries from a single study. If any study accounts for more than 10 papers or reports that study shall be included as a filter. The accompanying EGM report will identify the number of studies covered by the map and list those studies with multiple papers in an annex.

### 4.2 Presentation

The intervention and outcomes, described above, are the primary dimensions of the map.

In addition to intervention and outcomes, the following filters will be coded for primary studies (and reviews where appropriate).

1. Population subgroups of interest include: people who are sleeping rough; youth/young people; women; families with children/households with children; survivors of domestic violence/abuse; people who have experienced trafficking; LGBT; older people; discharged from health facilities; people with, or with history of, mental health problems/illness; people with alcohol or drugs issues; people with complex needs/dual diagnosis (e.g., alcohol and mental health issues); HIV positive; veterans/ex‐services; migrants (national and international)/non‐nationals; ex‐prisoners; people with disabilities; ethnic/racial minority; and rural areas.
2. Specific programmes and approaches: housing first, homeless veterans’ reintegration program, contingent approaches, noncontingent approaches.
3. Study (free text): where there are multiple papers from a single study.
4. Study designs: RCTs, natural experiments, regression discontinuity, propensity score matching, difference in difference, instrumental variables, other matching design.
5. Language of study: English, French, Spanish, Portuguese, Norwegian.
6. Global Region (World Bank categories)
7. Country
8. National region (e.g., state in the US, or country in UK such as England)
9. Language of study (English, French or Norwegian).

### 4.3 Planned analyses

The EGM report shall provide tabulations or graphs of the number of studies, with accompanying narrative description, by the following:

- intervention category and subcategory;
- outcome domain and sub‐domain;
- table of “aggregate map” of interventions and outcomes;
- region and country;
- year;
- study type.

The report will contain a network analysis of authors of included papers (see Rousseau, Egghe and Guns (2018): Chapter 10). In the network figure, each author will be represented by a circle, with size proportional to a number of studies authored, and lines connecting coauthors. The network will allow the identification of prominent authors and clusters of authors. (It will also help identify papers that are from a single study that may have been missed during coding.)

## 5 STAKEHOLDER ENGAGEMENT

The framework was developed through a consultative process.

Stage 1: Two existing frameworks were considered as a basis for the framework to be used for this map: (a) the intervention categories used by Munthe‐Kaas et al. (2016), and (b) the categories provided by Crisis (which are used in the SCIE, 2018, review).

Stage 2: The proposed framework was reviewed by the staff of Crisis and a group of UK academics specialising in homelessness (I‐SPHERE) and revised on the basis of their comments and further discussion with the Director of the new What Works Centre for Homelessness.

Stage 3: A group of homelessness researchers and practitioners reviewed the categories in an interactive exercise to fit the identified papers into the categories, resulting in further revision of those categories.

The map will be discussed with the Advisory Group for the Centre for Homeless Impact and presented at consultations organised by the Centre.

## ROLES AND RESPONSIBILITIES

Content: Ligia Teixeira and Suzanne Fitzpatrick. Ligia Teixeira is the Director of the new UK Centre for Homelessness Impact. Professor Suzanne Fitzpatrick has been researching homelessness for two decades with many scientific and official publications on the topic, and is an editor of the International Journal of Housing Policy.

Evidence gap methods: Howard White and Ashrita Saran, who have coauthored a paper on mapping methods used by different agencies. Howard White assisted the development of Campbell guidelines and standards for Evidence and Gap Maps.

Information retrieval: Ashrita Saran and Howard White. Ashrita Saran has received training on search strategies and authored strategies for other evidence synthesis products. The search strategy was adopted from that used by Munthe‐Kaas et al. (2016). The strategy was to be reviewed by John Eyres (IDCG Search Specialist) before submission. Audrey Portes will be part of the screening and coding team, and undertake searches for studies in French and Norwegian.

Project management: Audrey Portes will manage the project to ensure timely delivery.

## SOURCES OF SUPPORT

Production of the map has been supported by the UK Centre for Homelessness Impact with in‐kind support from the Campbell Collaboration Secretariat.

FHI is providing in‐kind support through the generous provision of all studies subject to full‐text screening for the review by Munthe‐Kaas et al. (2016).

## DECLARATIONS OF INTERESTS

Ligia Teixeira is the Director of the Centre for Homelessness Impact. This role should not provide any conflict as CHI's mission is to make evidence available. Suzanne Fitzpatrick is a leading researcher in the area so her some studies may be eligible for inclusion in the map.

## PRELIMINARY TIMEFRAME

Approximate date for submission of the EGM: November 8, 2018.

## PLANS FOR UPDATING THE EGM

The Centre for Homelessness Impact has agreed to provide resources to update the map every two years. The EGM team is in discussions with the EPPI Centre, who is responsible for the mapping software, about possible real‐time updating through (a) automated searches with machine‐learning powered screening and (b) moderated submissions of suggested papers.

## APPENDIX A SAMPLE SEARCH STRING

Search string/keywords (for ovid medline platform)

Study design key words

– (“quasi experiment*” or quasi‐experiment* or “random* control* trial*” or “random* trial*” or RCT or (random* adj3 allocat*) or matching or “propensity score” or PSM or “regression discontinuity” or “discontinuous design” or RDD or “difference in difference*” or difference‐in‐difference* or “diff in diff” or “case control” or cohort or “propensity weighted” or propensity‐weighted or “interrupted time series” or (before adj5 after) or (pre adj5 post) or ((pretest or pre test) and (posttest or post test)) or “research synthesis” or “scoping review” or “rapid evidence assessment” or “systematic literature review” or “Systematic review” or “Meta‐analy*” or Metaanaly* or “meta analy*” or “Control* evaluation” or “Control treatment” or “instrumental variable*” or heckman or IV or (quantitative or “comparison group*” or counterfactual or “counter factual” or counter‐factual or experiment*) adj3 (design or study or analysis)) or QED or evaluation).ti,ab,kw
– OR
– clinical trial/or clinical trial, phase i/or clinical trial, phase ii/or clinical trial, phase iii/or clinical trial, phase iv/or controlled clinical trial/or randomised controlled trial/or pragmatic clinical trial/
– controlled clinical trials as topic/or non‐randomised controlled trials as topic/or randomised controlled trials as topic/or pragmatic clinical trials as topic/or case‐control studies/or retrospective studies/or controlled before‐after studies/or interrupted time series analysis/or random allocation/or cohort studies/or follow‐up studies/or longitudinal studies/or prospective studies/or retrospective studies/or propensity score/or regression analysis/or evaluation studies/or matched‐pair analysis
– (“quasi experiment*” or quasi‐experiment* or “random* control* trial*” or “random* trial*” or RCT or (random* adj3 allocat*) or matching or “propensity score” or PSM or “regression discontinuity” or “discontinuous design” or RDD or “difference in difference*” or difference‐in‐difference* or “diff in diff” or “case control” or cohort or “propensity weighted” or propensity‐weighted or “interrupted time series” or (before adj5 after) or (pre adj5 post) or ((pretest or pre test) and (posttest or post test)) or “research synthesis” or “scoping review” or “rapid evidence assessment” or “systematic literature review” or “Systematic review” or “Meta‐analy*” or Metaanaly* or “meta analy*” or “Control* evaluation” or “Control treatment” or “instrumental variable*” or heckman or IV or ((quantitative or “comparison group*” or counterfactual or “counter factual” or counter‐factual or experiment*) adj3 (design or study or analysis)) or QED).ti,ab,kw.

(“meta regression” or “meta synth*” or “meta‐synth*” or “meta analy*” or “metaanaly*” or “meta‐analy*” or “metanaly*” or “metaregression” or “metaregression” or “methodologic* overview” or “pool* analys*” or “pool* data” or “quantitative* overview” or “research integration”).ti,ab,sh.

OR

(review adj3 (effectiveness or effects or systemat* or synth* or integrat* or map* or methodologic* or quantitative or evidence or literature)).ti,ab,sh.

### Homelessness keywords

– homeless persons/or homeless youth
– (evict* or homeless* or "housing excl*" or “residential stability” or ((street* or private or improvised or shelter* or emergency or temporary or insecure or overcrowded or precarious or stable or marginal*) adj3 (dwell* or house* or housing or accommodation)) or (street adj3 (life or living or lives or youth* or child* or people or person*)) or runaway* or “Run away from home” or “Running away” or “Ran away” or “Going missing” or “Bag lady” or Houseless* or Unhoused or “without a roof” or Roofless or (rough adj3 sleep*) or Destitut* or “Skid row*” or “sleepers out”).ti,ab,kw.
– (“Housing first” or “Pathways to Housing” or “Homeless Veterans Reintegration Program” or “Access to Community Care and Effective Services and Supports” or 'Support* Housing Program” or “Housing and Urban Development–Veterans Affairs Supported Housing program” or “HUD‐VASH” or “Sober Transitional Housing and Employment Project” or “sober house placement*” or “Housing ladders” or “Staircase housing” or “low threshold housing” or “Critical Time Intervention”).ti,ab,kw.

## APPENDIX B KEY ITEMS FROM CODING FORM

### Study characteristics

1. Study design
  1.1. Systematic review
  1.2. RCT
  1.3. Natural experiments
  1.4. Quasi‐experimental
  1.5. Cohort
  1.6. Cross sectional
  1.7. Mixed Method
  1.8. Other matching design
2. Status of study
  2.1. Completed
  2.2. Ongoing
3. Systematic review quality
  3.1. Low
  3.2. Moderate
  3.3. High
  3.4. Not applicable (Primary study)
4. Study region
  4.1. East Asia and Pacific
  4.2. Europe and Central Asia
  4.3. Latin America & the Caribbean
  4.4. Middle East and North Africa
  4.5. North America
  4.6. South Asia
  4.7. Sub‐Saharan Africa
  4.8. National Region: state in the US, or country in UK such England
5. Population
  5.1. Youth/young People; women
  5.2. Families with children/Households with children
  5.3. Survivors of domestic violence/abuse
  5.4. LGBT
  5.5. Older people
  5.6. Discharged from health facilities
  5.7. People with, or with history of, mental health problems/illness
  5.8. People with alcohol or drugs issues; people with complex needs/dual diagnosis (e.g. alcohol and mental health issues)
  5.9. HIV positive
  5.10. Veterans/Ex‐services
  5.11. Migrants (national and international)/Non‐nationals
  5.12. Ex‐prisoners
  5.13. People with disabilities
  5.14. Rural areas
6. Intervention
  6.1. Legislation
    6.1.1 Housing/homelessness/vagrancy legislation/act
    6.1.2 Welfare/benefits legislation (universal credit)
    6.1.3 Social and health care legislation
  6.2. Prevention
    6.2.1. Primary
    6.2.2. Secondary
    6.2.3. Tertiary
  6.3. Services and Outreach
    6.3.1. Soup runs and soup kitchens
    6.3.2. Day centres/centres
    6.3.3. Outreach/street workers (including traditional street outreach)
    6.3.4. Assertive street outreach (No second night out, no third night out)
    6.3.5. Reconnection and relocation
  6.4. Accommodation‐Based Interventions
    6.4.1. Shelters and hostels
    6.4.2. Supported accommodation
    6.4.3. Social/public housing
    6.4.4. Private rented housing including shared tenancies
    6.4.5. Community hosting
  6.5. Employment
    6.5.1. Supported employment and individual placement schemes/support
    6.5.2. Mentoring and coaching (including job coaching)
    6.5.3. Flexible employment options
  6.6. Health and social care
    6.6.1. Health interventions (primary, secondary and tertiary)
    6.6.2. Substance misuse treatment (residential rehab/detox, community based)
    6.6.3. Mental health services (drugs, CBT, etc.)
    6.6.4. Specialist homelessness health services
    6.6.5. Case workers
  6.7. Education and skills
    6.7.1. Vocational training
    6.7.2. Work experience (including volunteering)
    6.7.3. Life skills training (including rights)
    6.7.4. Education for homeless children
    6.7.5. Creative activities
  6.8. Communication
    6.8.1. Advocacy campaigns (no one turned away)
    6.8.2. Public information campaigns
    6.8.3. Health Promotion
7. Outcomes
  7.1. Access to services
    7.1.1. Health care (primary, secondary and tertiary care)
    7.1.2. Welfare benefits
    7.1.3. Other services
  7.2. Justice (including criminalisation)
    7.2.1. Arrest and imprisonment
    7.2.2. Recidivism
    7.2.3. Victims of crime
  7.3. Housing stability
    7.3.1. Accommodation/housing status
    7.3.2. Satisfaction with housing
  7.4. Health (inc substance abuse)
    7.4.1. Abstinence from substance abuse
    7.4.2. Physical health and nutrition status
    7.4.3. Mental health status
  7.5. Employment and income
    7.5.1. Employment status (paid and unpaid work)
    7.5.2. Earned income
    7.5.3. Forced labour/labour and sex work
  7.6. Capabilities & Wellbeing
    7.6.1. Improved skill and self‐care
    7.6.2. Community engagement and social connectedness.
    7.6.3. Overall wellbeing/quality of life.
  7.7. Public attitudes and participation
    7.7.1. Public understanding of homelessness
    7.7.2. Support for interventions for homelessness
    7.7.3. Fundraising
    7.7.4. Public engagement in homelessness‐related activities
  7.8. Cost
    7.8.1. Cost effectiveness
    7.8.2. Savings
    7.8.3. Cost per participant
8. Language
  8.1. English
  8.2. French
  8.3. Spanish
  8.4. Portuguese
  8.5. Norwegian

## APPENDIX C

**Table C1:** Critical appraisal tool for primary studies

Item		Point in time (where applicable)	Rating
1a	Study design (potential confounders taken into account)	End of intervention	High confidence: RCT, RDD, ITT, instrumental variable
			Medium confidence: DiD with matching, PSM
			Low confidence: other matching
1b	Study design (potential confounders taken into account)	Longest follow up (if applicable)	Study design may change at post endline follow up, usually loss of RCT as control becomes treated. Same codes as 1a
2	Masking or blinding (RCTs only)		High confidence: any blinding or any mention of blinding
			Medium confidence: no blinding
			Low confidence is not used for this item
3	Power calculations are reported		High confidence: any mention of power calculations as basis for sample size
			Medium confidence: no mention of power calculations
			Low confidence is not used for this item
4a	Losses to follow up are presented and acceptable^a^	End of intervention	High: attrition within IES bounds
			Medium: attrition within IES liberal bounds
			Low: attrition not reported or attrition outside IES bounds
			N/A for ex post studies
4b	Losses to follow up are presented and acceptable^a^	Longest follow‐up (if applicable)	High: attrition within IES bounds
			Medium: attrition close to IES bounds
			Low: attrition not reported or attrition outside IES bounds
			N/A for ex‐poststudies
5	Intervention if clearly defined		High confidence: intervention clearly and fully described
			Medium confidence: brief description of intervention
			Low confidence: intervention named but not described, or not named
6	Outcome measures are clearly defined and reliable		High confidence: outcome measure clearly and fully described, preferably with reference to validation
			Medium confidence: brief description of outcome
			Low confidence: outcome named but not described
7	Baseline balance (N.A. for before versus after)		High confidence: RCT or baseline balance report and satisfactory (imbalance on 2 or less measures)
			Medium confidence: imbalance on no more than 5 measures
			Low confidence: baseline balance not reported, or reported and lack of balance on more than 5 measures
	Overall confidence in study findings	End of intervention	Lowest rating across items 1a, 4a, 6 and 7
	Overall confidence in study findings	Longest follow up (if applicable)	Lowest rating across items 1b, 4b, 6 and 7 (N/A if 1b and 4b N/A)

^a^ See Table 1 https://homvee.acf.hhs.gov/HomVEE‐Attrition‐White_Paper‐7–2015.pdf
